# Disentangling the Effect of BMI on Hepatocellular Carcinoma From Cirrhosis With Multivariable Mendelian Randomization

**DOI:** 10.1111/liv.70609

**Published:** 2026-03-19

**Authors:** Apostolos Gkatzionis, Eleanor Sanderson, George Davey Smith, Stefan Stender, Helene Gellert‐Kristensen

**Affiliations:** ^1^ MRC Integrative Epidemiology Unit, Population Health Sciences University of Bristol Bristol UK; ^2^ Department of Clinical Biochemistry Copenhagen University Hospital – Rigshospitalet Copenhagen Denmark; ^3^ Department of Clinical Medicine, Faculty of Health and Medical Sciences University of Copenhagen Copenhagen Denmark

**Keywords:** childhood obesity, European population, fibrosis, metabolic dysfunction‐associated steatotic liver diseases, obesity

## Abstract

**Background & Aims:**

While cirrhosis is a primary risk factor for hepatocellular carcinoma (HCC), a significant proportion of HCC cases attributed to metabolic dysfunction‐associated steatotic liver disease (MASLD) develop in the absence of cirrhosis. MASLD is strongly linked to obesity, a known risk factor for multiple cancers. Whether the effect of obesity on HCC is mediated via cirrhosis or other factors is unknown.

**Methods:**

We used univariable Mendelian randomization (MR) to test the total effect of a higher body mass index (BMI), a proxy for obesity, on HCC, and multivariable MR to test the direct effect.

**Results:**

We estimated that the effect of BMI was a 1.65‐fold higher risk of HCC per standard deviation increase (95% confidence interval (CI): 1.28–2.12, *p*‐value = 1.0 × 10^−5^). The BMI effect became indistinguishable from zero when taking liability to cirrhosis into account with multivariable MR (odds ratio = 1.12, 95% CI: 0.84–1.50, *p*‐value = 0.44). We investigated additional potential pathways linking BMI to HCC—such as inflammation and type 2 diabetes—and explored the direct effect of childhood obesity on the risk of HCC. We found no direct effect of inflammation or type 2 diabetes (*p*‐values > 0.05). Childhood body size increased the risk of HCC (odds ratio = 1.78, 95% CI: 1.27–2.49, *p*‐value = 8 × 10^−4^), but the effect disappeared when we took adult body size into account using multivariable MR.

**Conclusions:**

Cirrhosis liability is the primary mediator of the causal effect of obesity on HCC.

## Summary

Obesity significantly raises the risk of liver cell cancer. Using genetic evidence, our results show that the likelihood of developing cirrhosis (scarring of the liver) mediates the entire risk of liver cell cancer seen with obesity. This suggests that preventing liver scarring is the key to stopping obesity‐related liver cell cancer and urges further investigation into alternative causes for obese patients who develop liver cell cancer without cirrhosis.

**Abbreviations**
BMI: body mass index
CI: confidence interval
CRP: c‐reactive protein
EUR: European
GWAS: genome‐wide association study
HbA1C: haemoglobin A1C
HCC: hepatocellular carcinoma
HOMA‐B: homeostasis model assessment of β‐cell function
IL‐1R antagonist: interleukin‐1‐receptor antagonist
IL‐6: interleukin‐6
IL‐6R subunit alpha: Interleukin‐6 receptor subunit alpha levels
IVW: inverse‐variance weighted
MASLD: metabolic dysfunction‐associated steatotic liver disease
MR: Mendelian randomization
MVMR: Multivarimble Mendelian randomization
OR: odds ratio
RNA: ribonucleic acid
SD: standard deviation
SNP: single nucleotide polymorphism
SUGP1: SURP and G‐patch domains‐containing protein 1
TM6SF2: transmembrane 6 superfamily member 2

## 1 Introduction

Metabolic dysfunction‐associated steatotic liver disease (MASLD)—historically called non‐alcoholic fatty liver disease—affects 30% of the adult population [1]. The disease is closely related to obesity, and its 50% rise in prevalence over the past three decades mirrors that of obesity [1, 2]. MASLD is a spectrum of liver abnormalities. The initial stage is an accumulation of excess fat (steatosis), which can lead to inflammation of the liver tissue (steatohepatitis) complicated by scarring of the liver (fibrosis) that can eventually amass to cirrhosis. Approximately 1% of patients with MASLD progress to cirrhosis [3], which is a major risk factor for developing hepatocellular carcinoma (HCC), a cancer with an 18% five‐year survival rate [4]. Alarmingly, the number of new HCC cases is predicted to double from 2022 to 2050 [5].

HCC can occur in the absence of cirrhosis, and does so in 22%, 6%, 9%, and 39% of HCC cases attributed to hepatitis B virus infection, hepatitis C virus infection, alcohol‐associated liver disease, and MASLD, respectively [6, 7, 8]. The higher frequency of non‐cirrhotic HCC for patients with hepatitis B is hypothesized to occur because the virus integrates itself into the host genome, altering oncogene expression in the process [9]. What drives the high degree of malignant transformation in non‐cirrhotic MASLD is less clear. Obesity, the major predisposing factor for MASLD, is a known risk factor for multiple cancers, including HCC, with low‐grade inflammation and insulin resistance suggested as mechanistic links [10, 11, 12].

Whether obesity has direct effects on HCC, independent of its risk‐increasing effect on cirrhosis, is not known. This is a clinically important question because if obesity has a direct effect on HCC, weight‐loss interventions could reduce HCC risk beyond lowering the risk of cirrhosis. However, answering this question is difficult using traditional methods. Randomised controlled trials would be logistically unfeasible due to immense sample sizes and long follow‐up times, and a weight‐gain trial would be unethical. Furthermore, observational studies on this topic are susceptible to confounding and reverse causation.

To address these challenges, we employed Mendelian randomization (MR), an epidemiological approach that uses genetic variants as proxies, thereby minimising confounding and strengthening causal inference [13].

**FIGURE 1:**
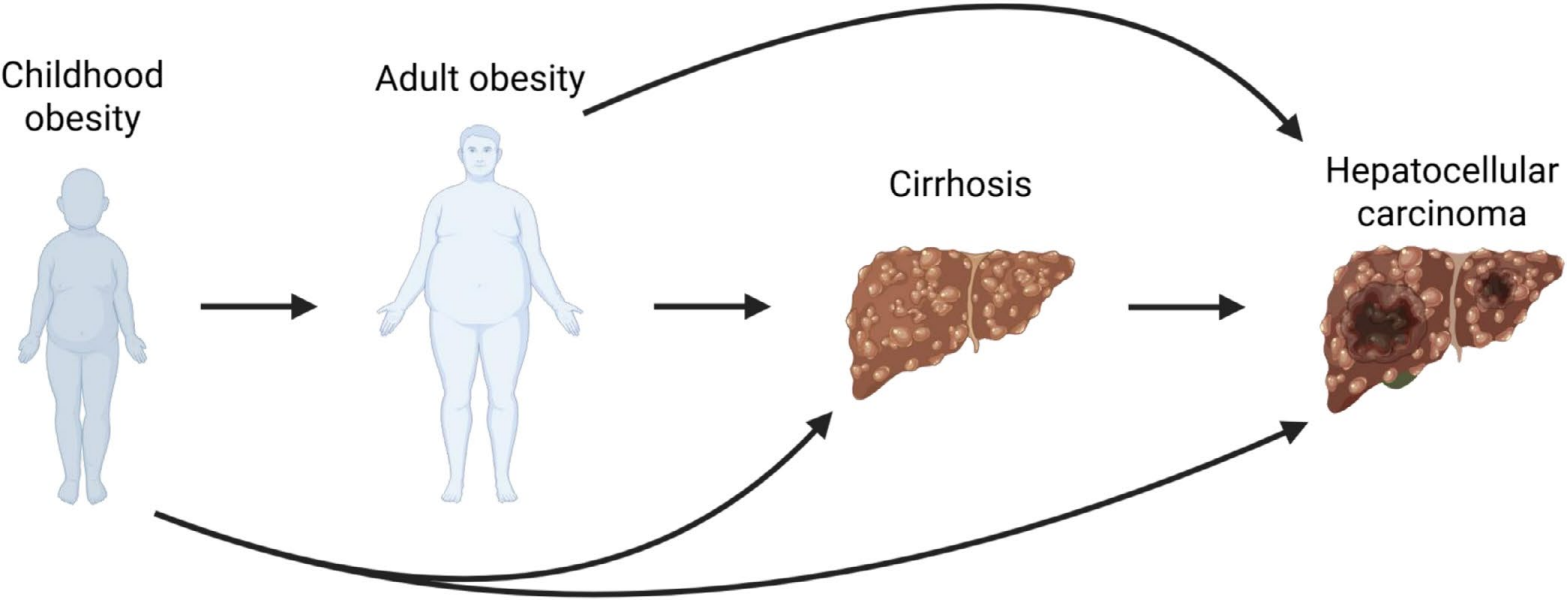
Directed acyclic graph of the conceivable causal relationship between obesity, cirrhosis, and hepatocellular carcinoma. Obesity is proxied with body mass index. The arrows signify potential pathways based on associations from observational studies and clinical trials. Figure created with Bio Render.

We hypothesized that the effect of BMI on HCC could be disentangled from that of cirrhosis using multivariable Mendelian randomization (MVMR). Using two recently published, large genome‐wide association studies (GWAS) of cirrhosis [14] and HCC, we first assessed the total causal effect of BMI on HCC using univariable MR. We then used MVMR to examine the direct causal effect of BMI on HCC while accounting for cirrhosis as a mediator (Figure 1). We also investigated mechanistic pathways potentially linking BMI to HCC and explored the impact of childhood obesity on the risk of HCC.

## 2 Methods

### 2.1 Genetic Instruments From Genome‐Wide Association Study Summary Statistics

Genetic instruments for MR were created from GWAS summary statistics from published articles or openGWAS [15, 16]. To limit potential confounding by ancestry, we used GWAS results derived from individuals of European descent. When more than one GWAS‐significant (*p*‐value < 5 × 10^−8^) single nucleotide polymorphism (SNP) existed within a 10 000 base pair window, we clumped them together using a linkage disequilibrium *R* ^2^ < 0.001 in the 1000G phase 3 EUR‐population as a cut‐off.

The key exposures were: BMI transformed into units of standard deviation [17] and cirrhosis on a liability scale in log (odds) units [18]. We used data from the largest available GWAS of HCC as our outcome [19]. The full list of summary statistics used as exposures is provided in Table S1.

### 2.2 Total Effect Estimates With Univariable Mendelian Randomization Analysis

The total causal effect is the effect of an exposure on an outcome through all pathways that are consequences of the exposure, whether direct or mediated. We used the R package TwoSampleMR [16] to perform univariable MR on summary statistics to estimate the total causal effect. The main analysis was a multiplicative random effects meta‐analysis weighted by the inverse of the standard error of the SNP‐specific exposure‐outcome causal effect estimate squared and with correction for under‐dispersion—the default inverse variance‐weighted (IVW) analysis in the TwoSampleMR package at the time of this study. In the univariable analysis of BMI on HCC, the F‐statistic was used to assess the potential for weak instrument bias. We calculated the statistic by dividing the mean value of the SNPs' beta estimate squared with the SNPs' standard error squared. An F‐statistic equal to or above ten designated the genetic instrument sufficiently strong for causal inference [20]. The IVW Q‐statistic was calculated to look for heterogeneity. A Q‐statistic value above the number of genetic instruments suggests heterogeneity and was followed up with a scatterplot and MR‐Egger analysis. MR‐Egger regresses SNP‐outcome on SNP‐exposure summary statistics, including an intercept in the regression model, which tests and corrects for directional pleiotropy in the causal estimate [21]; the method provides consistent causal effect estimates under the InSIDE assumption, but potentially at the expense of power [20]. We considered an MR‐Egger intercept with a large estimate or a *p*‐value < 0.05 indicative of directional horizontal pleiotropy in the genetic instruments, which is a violation of the exclusion restriction assumption.

In addition, we conducted weighted median and weighted mode analyses as sensitivity analyses of BMI's effect on HCC. The weighted median is robust to individual genetic variants with strong outlying effects and yields pleiotropy‐robust causal estimates under the assumption that more than 50% of the weight of the instruments comes from valid instruments [22]. The weighted mode provides robust estimates when the largest weights among the 𝑘 subsets are contributed by valid instruments (i.e., the zero modal pleiotropy assumption) [23]. Our belief that causal inference is reliable was strengthened if estimates were consistent between the MR‐IVW, MR‐Egger, MR weighted median, and MR weighted mode.

### 2.3 Direct Effect Estimates With Multivariable Mendelian Randomization Analysis

The direct causal effect is the consequence of an exposure on an outcome that is not mediated through other exposures included in the model. MVMR assesses multiple exposures on an outcome simultaneously and conditional on each other [24]; the resulting exposure estimates are direct effect estimates. To attain the combined genetic instrument for MVMR, we aggregated the exposure of interest (obtained as described in the univariable Mendelian Randomization section) and clumped the SNPs once again based on linkage disequilibrium. We excluded any SNPs for which the summary statistics were unavailable in the exposures or outcome GWAS.

We utilised the R package MVMR to perform the analyses [25]. The main analysis consisted of an IVW MVMR model (MVMR‐IVW) [24]. Instrument strength was assessed for each exposure in the model using conditional F‐statistics. If the F‐statistics for all exposures are above ten, the bias from weak instruments is likely to be minimal [25]. We calculated a modified version of Cochrane's Q (Qa‐statistic) with a corresponding *p*‐value to test for pleiotropy in the combined genetic instrument [25]. A threshold of *p*‐value < 0.05 was considered indicative of pleiotropy in the MVMR genetic instrument. Since sample overlap between exposures in the MVMRs cannot be excluded, we used the summary statistics and the metaCCA R‐package [26] to calculate the phenotypic correlation matrix for exposures BMI, cirrhosis, alcohol, diabetes mellitus type II, childhood body size, and adult body size to account for the covariance of genetic instruments in the calculation of the conditional F and Qa statistics. We used a covariance of zero when the phenotypic correlation could not be calculated.

As sensitivity analyses, we inspected the fitted vs. residual values plot for the MVMR‐IVW analysis to look for outlying SNPs. We also conducted pleiotropy robust analyses using MVMR‐robust and MVMR‐median, which provide robustness to influential outliers and directional pleiotropy, respectively [27]. Our belief that causal inference is reliable was strengthened if estimates were consistent between the MVMR‐IVW, MVMR‐robust, MVMR‐median, and between models including additional risk factors correlated with BMI and suggested to influence the risk of HCC (alcohol consumption, CRP, IL‐1R antagonist, IL‐6, and IL‐6R alpha subunit, waist‐to‐hip ratio, diabetes mellitus type II, HbA1C, and HOMA‐B, and educational attainment as a proxy for socioeconomic status).

### 2.4 Time‐Varying Genetic Instruments

Childhood and adult obesity are correlated because obese children tend to remain large in adulthood [28]. To untangle the two, we used GWAS data of childhood and adult body size from UK biobank extracted from OpenGWAS as proxies [29]. Childhood body size is an ordered categorical variable of self‐reported comparative body size at age 10, where participants were asked if When you were 10 years old, compared to average would you describe yourself as thinner, plumper, or about average? The exposure was validated against BMI at mean age 10 and 18 in the Avon Longitudinal Study of Parents and Children [29] and BMI at age 12–15 and in the Norwegian Trøndelag Health Study [30]. Adult body size is an ordered categorical variable created from BMI (kg/m^2^) to match the categories available from childhood body size. The instruments are suitable for unravelling childhood obesity from adult obesity [29, 31]. We conducted Mendelian randomization to estimate the total and direct effects of childhood and adult body size as described earlier in this method section.

## 3 Results

### 3.1 The Total Effect of BMI on HCC

**FIGURE 2:**
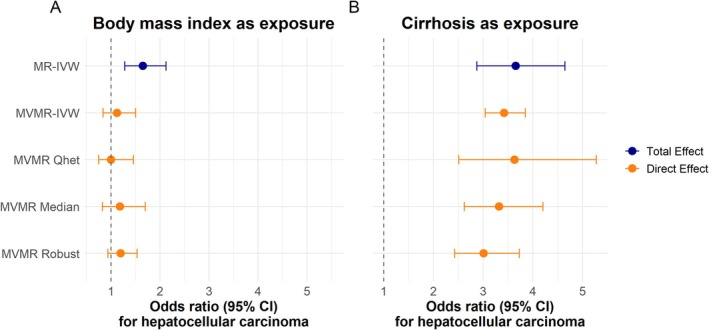
The total and direct causal effect estimates of BMI and cirrhosis on hepatocellular carcinoma. Panels A and B show the results for BMI and cirrhosis for different Mendelian randomization methods. BMI is reported in units of standard deviation. Dots are point estimates, and error bars are 95% CIs. The blue dot is the estimate from a univariable MR using the standard IVW‐method. The orange dots are the direct effect, meaning the causal effect on hepatocellular carcinoma in MVMR including both BMI and cirrhosis. The MVMR‐IVW estimate is the main multivariable analysis while the Qhet, Median, and Robust methods are sensitivity analyses. BMI: Body mass index. CI: Confidence interval. MR: Mendelian randomization. MVMR: Multivariable Mendelian randomization. IVW: Inverse variance‐weighted.

We first estimated the total causal effect of BMI on the risk of HCC. BMI affected HCC with an odds ratio (OR) of 1.65 per standard deviation (SD) increase (95% CI: 1.28–2.12, *p*‐value = 1 × 10^−5^) in the MR‐IVW analysis (Figure 2, panel A). The genetic instruments exhibited heterogeneity (Q‐statistic = 177 for 140 instruments, Figure S1), which is well balanced (Egger intercept beta = −3 × 10^−5^, *p*‐value = 1.00). The analysis had sufficient strength for causal inference (F‐statistic = 42). Sensitivity analyses yielded similar estimates to the MR‐IVW (Table S2).

### 3.2 The Direct Effect of BMI and Cirrhosis on HCC

We hypothesized that cirrhosis was the primary mediator of the effect of obesity on HCC. We therefore calculated the direct effect of BMI and cirrhosis liability on the risk of HCC with MVMR‐IVW. Unsurprisingly, cirrhosis had a causal direct effect on HCC with an OR of 3.42 per log odds increase in genetic liability to cirrhosis (95% CI: 3.05–3.84, *p*‐value = 1 × 10^−44^)—similar to the total effect from MR‐IVW—with congruent estimates in the MVMR‐IVW, MVMR‐robust, and MVMR‐median (Figure 2, panel B). In contrast, we did not find a direct effect of BMI on HCC (OR = 1.12 per SD increase, 95% CI: 0.84–1.50, *p*‐value = 0.44) with the sensitivity analyses recapitulating the absence of a direct effect of BMI on HCC (Figure 2, Panel A).

The instruments had adequate strength for causal inference (conditional F‐statistic: BMI = 58 and cirrhosis = 16) but exhibited pleiotropy (*p*‐value for the Qa statistic = 0.001) (Table S3). We inspected the residual vs. fitted plot from the MVMR‐IWV to look for influential outliers and identified two variants—rs739846 and rs188247550, both located in the RNA splicing‐related gene *SUGP1* near the known MASLD‐locus *TM6SF2*– with large fitted and residual values (Figure S2). The variants had negligible effects on BMI but substantial effects on cirrhosis and HCC (Table S4). When we excluded the two variants from the analysis, the resulting estimates were comparable to the MVMR‐IVW estimates including all variants, and heterogeneity diminished (*p*‐value for the Qa statistic = 0.07) (Table S5).

### 3.3 Potential Independent Risk Factors for HCC

**FIGURE 3:**
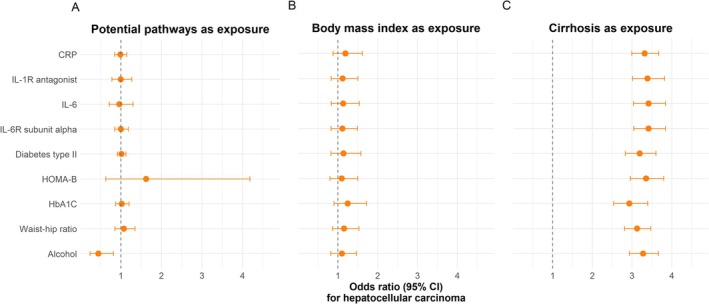
The direct causal effect estimates of BMI, cirrhosis, and potential mechanistic pathways on hepatocellular carcinoma. Panels A, B, and C show the results for the potential mechanistic pathways, BMI, and cirrhosis, respectively. The estimates are from MVMR‐IVW analyses including BMI, cirrhosis, and potential mechanistic pathways as exposures and hepatocellular carcinoma as the outcome. The potential pathways were included one at a time, and each row represents estimates from the MVMR‐IVW analysis including that genetic instrument. Dots are point estimates of the direct effect, and error bars are 95% CIs. BMI: Body mass index. CI: Confidence interval. CRP: C‐reactive protein. HbA1c: Haemoglobin A1c. HOMA‐B: Homeostatic model assessment of beta‐cell function. IL‐1R: Interleukin‐1 receptor. IL‐6R: Interleukin‐6 receptor. IVW: Inverse variance‐weighted. MVMR: Multivariable Mendelian randomization.

We examined additional risk factors correlated with BMI and suggested to influence the risk of HCC. We tested inflammation (CRP, IL‐1R antagonist, IL‐6, and IL‐6R alpha subunit), diabetes mellitus (diabetes mellitus type II, HbA1C, and HOMA‐B), waist‐hip ratio, and alcohol consumption (Table S1). Each risk factor was included, one at a time, as the third exposure in an MVMR‐IVW of BMI and cirrhosis on HCC (Figure 3, Panel A). We found no direct effect of inflammation, diabetes mellitus, or waist‐to‐hip ratio on the risk of HCC (Figure 3, Panel A). HOMA‐B had a large effect estimate but a very wide confidence interval overlapping one. We found a direct effect of alcohol with an OR of 0.44 per increase in the number of weekly drinks (95% CI: 0.24–0.82, *p*‐value = 0.01).

For six of the nine analyses, the MVMR showed evidence of weak instrument bias, and for eight of the nine, the *p*‐value for the Qa statistic indicated potential pleiotropy (Table S6). However, the direct effect estimates of cirrhosis and BMI on HCC were consistent across the models and with the results from the previous MVMR estimates (Figure 3, Panels B and C). In sensitivity analyses using MVMR Median and MVMR Robust, the effect of alcohol was lessened with confidence intervals overlapping one (Table S7).

### 3.4 Direct Effects of Childhood and Adult Body Size on Cirrhosis and HCC

**FIGURE 4:**
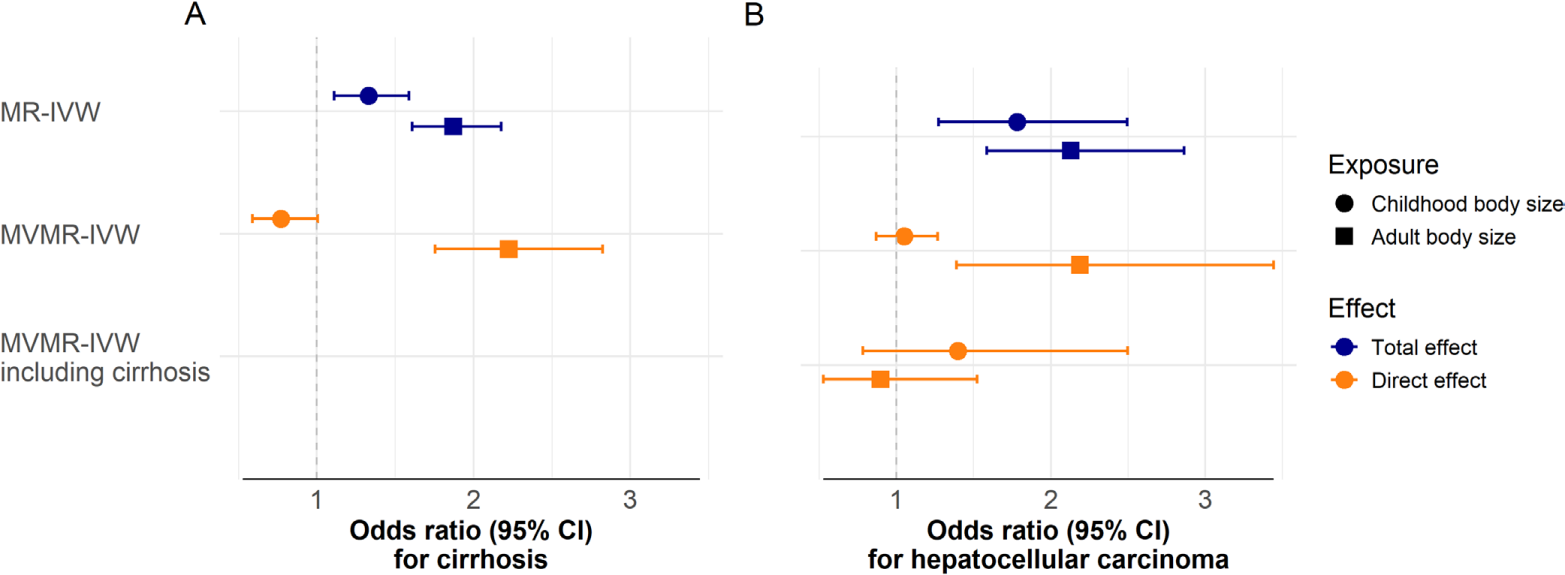
The total and direct causal effect of childhood and adult body size on cirrhosis and hepatocellular carcinoma. Panels A and B show the results from MR and MVMR analyses using cirrhosis and hepatocellular carcinoma as the respective outcomes. The genetic instrument for childhood body size is derived from a genome‐wide association study of childhood body size and has been validated against childhood BMI in two separate cohorts. The genetic instrument for adult body size is based on BMI measurements, which were converted to body size categories for the genome‐wide association study. These genetic instruments have been used to separate childhood body size from adult body size in previous studies. The methods used were MR‐IVW analyses of childhood and adult body size (the total effect), MVMR‐IVW analyses with childhood and adult body size as exposures (direct effect), and MVMR‐IVW analysis with childhood body size, adult body size, and cirrhosis as exposures (direct effect). Dots are point estimates, and error bars are 95% CIs. BMI: Body mass index. CI: Confidence intervals. MR: Mendelian randomization. MVMR: Multivariable Mendelian randomization. IVW: Inverse variance‐weighted.

We aimed to disentangle the effects of childhood body size from adult body size on cirrhosis and HCC. In MR‐IVW, both childhood and adult body size increased the risk of HCC and cirrhosis (Figure 4, Panel A and B, Table S8). When we included both exposures in an MVMR‐IWV analysis, we found that adult body size had an OR of 2.23 (95% CI: 1.75–2.82, *p*‐value = 2 × 10^−10^) and 2.19 (95% CI: 1.39–3.44, *p*‐value = 8 × 10^−4^) per category increase for cirrhosis and HCC, respectively, while childhood body size showed no direct effect when adult body size was accounted for (cirrhosis OR = 0.77 per categories of body size, 95% CI: 0.59–1.01, *p*‐value = 0.06; and HCC OR = 1.10 per category of body size, 95% CI: 0.66–1.82, *p*‐value = 0.71). Additionally, when cirrhosis was accounted for body size did not affect the risk of HCC.

There was no indication of weak instrument bias in the MR‐IVW analyses (all F‐statistics > 10), and while Q statistics suggested heterogeneity in the analyses (all *p* values for Q statistic < 0.05), it did not appear to cause directional horizontal pleiotropy (All MR‐Egger intercept *p*‐values > 0.05). In the MVMR‐IVW including childhood and adult body size, the instruments had adequate strength with conditional F‐statistics of 15 and 17, respectively. When adding cirrhosis to the MVMR‐IVW as an exposure, the F‐statistics were 12, 13, and 7.6 for childhood body size, adult body size, and cirrhosis liability, respectively. There was no substantial heterogeneity in the per‐SNP effect estimates for cirrhosis and HCC, respectively (Tables S8–S11).

## 4 Discussion

The main finding of this MVMR study is that liability to cirrhosis fully explains the risk‐increasing effect of obesity on HCC. Our study is the first aimed at disentangling the HCC‐promoting effect of obesity from that of cirrhosis in an MR framework. Previous observational epidemiological studies have firmly established a link between obesity and HCC. A meta‐analysis of 22 studies with 6 059 561 individuals found that a BMI above 35 kg/m^2^ is associated with a 3‐fold increased risk of primary liver cancer (of which HCC constitutes 90%) [32]. In univariable analyses, we found genetically proxied higher BMI to be robustly associated with higher HCC risk, with a 1‐SD higher BMI conferring a 65% increased risk of HCC. When using MVMR to account for cirrhosis, the effect of BMI on HCC was considerably reduced and became statistically nonsignificant. We further tested the inclusion of various markers of inflammation and insulin resistance in the MVMR analysis. These analyses yielded no significant direct effects for inflammation or insulin resistance, supporting that low‐grade inflammation or insulin resistance either have little direct role beyond fibrogenesis in the development of obesity‐associated HCC or that any such effect is secondary to fibrosis. The direct effect estimates of BMI and cirrhosis remained unchanged when taking inflammation and insulin resistance into account. Taken together, these analyses support a model in which obesity mediates its effect on HCC entirely via cirrhosis.

While observational studies show that a significant proportion of individuals diagnosed with MASLD and HCC lack advanced fibrosis or cirrhosis, the reason for this is not fully understood [33]. Given the high prevalence of MASLD and metabolic syndrome in the population, it is likely that other risk factors often coexist with MASLD. Several known causes, including alcohol, can also mimic or worsen MASLD [34], each carrying a different risk for HCC [35]. Therefore, for non‐cirrhotic and non‐fibrotic HCC patients, further investigation into coexisting factors such as toxic exposures [36, 37] and genetic diseases [38, 39, 40, 41] may be warranted.

Childhood obesity has been observed to increase the risk of primary liver cancer in adulthood [42]. However, childhood obesity often persists into adulthood, making it difficult to separate the two [28]. In our MR‐IVW analysis, we found that an increase in childhood body size conferred a 78% higher risk of HCC in adulthood. However, after conditioning on adult body size, the direct effect of childhood body size disappeared. Additionally, when accounting for cirrhosis, neither childhood nor adult body size had a direct effect on HCC. Taken together, these results are consistent with previous reports that the effect of childhood obesity on the risk of coronary artery disease and type 2 diabetes later in life can be attributed to individuals remaining large into adulthood [29].

This study has several limitations. First, modelling complex, time‐varying and/or behavioural exposures like BMI, alcohol consumption, inflammation, and insulin resistance using genetic proxies in an MVMR framework relies on several assumptions and is inherently a simplification. Each estimate should therefore be interpreted cautiously and compared to existing evidence, where possible. For example, the MVMR analysis that included alcohol, BMI, and cirrhosis in the model suggested a decreased risk of HCC with higher alcohol intake, a nonsensical result given the known causal role of alcohol on HCC. Indeed, we have previously shown that genetically proxied alcohol consumption robustly increases the risk of HCC in univariable MR [19]. The apparent protective effect of alcohol in the MVMR analysis likely reflects an artefact and not a true effect [43]. Potential sources of the artefact include: Competing risk of death in heavy alcohol consumers before a diagnosis of HCC (particularly in individuals with cirrhosis) [44]; discouragement of alcohol consumption in patients with early signs of liver disease; measurement error in self‐reported alcohol consumption, where individuals with higher alcohol consumption have a tendency to under‐report [36, 45]; and index event bias where individuals with a higher alcohol consumption are less likely to participate in research. Second, the residual nonsignificant direct effect estimate for BMI across MVMRs could, in theory, reflect a smaller but genuine effect of BMI. Given that cirrhosis is a binary endpoint of fibrosis, and that advanced fibrosis is frequently present in non‐cirrhotic MASLD‐HCC patients [46], it is more likely that fibrosis explains this residual effect. Third, BMI is an imperfect proxy for obesity, with waist‐hip ratio suggested as an alternative [47]. When we included waist‐hip ratio in the MVMR, it had no direct effect, nor did it change the BMI and cirrhosis estimates. Fourth, low conditional F‐statistics and heterogeneity in the MVMRs affected confidence in the exclusion‐restriction and independence assumptions. However, consistent estimates for cirrhosis and BMI across methods and models suggest these findings are robust. The results apply to the population as a whole, but whether results differ depending on genetic vulnerability to liver disease (e.g., carriership of *PNPLA3* p.Ile148Met) was not examined. Fifth, there are limitations related to the cirrhosis definitions used in the study cohorts. The cirrhosis GWAS included nine European cohorts with > 90% of case definitions based on ICD codes [18]. An inherent limitation to this is that some degree of misclassification is inevitable. That said, cirrhosis is a hard clinical endpoint with well‐defined diagnostic criteria. Another limitation related to the cirrhosis definition is that cirrhosis may in some individuals be attributed to the presence of HCC (i.e., detection bias). Previous studies from Sweden and the US found that a concomitant diagnosis of HCC and cirrhosis occurs in 39%–57% of HCC cases [48, 49]. Sixth, we did not test other proposed links between obesity and cancer (altered sex hormone metabolism, altered IGF signalling, microbiome) [11, 12], and can therefore not conclude whether these affect HCC independent of cirrhosis. As BMI itself did not have a clear direct effect on HCC risk, these alternative pathways are unlikely to underlie the high prevalence of non‐cirrhotic MASLD‐HCC.

In conclusion, we found that obesity's causal effect on HCC is mediated entirely by cirrhosis liability. We saw that the higher risk of HCC in obese children can be attributed to individuals remaining larger into adulthood. We did not find evidence supporting low‐grade inflammation or insulin resistance as mechanistic links between obesity and HCC when cirrhosis was accounted for. These results suggest obesity per se is not the cause of the high number of non‐cirrhotic MASLD‐related HCC. Further investigation into other causes may be warranted for this group of patients.

## Author Contributions

Helene Gellert‐Kristensen, Stefan Stender, George Davey Smith, Apostolos Gkatzionis, and Eleanor Sanderson conceived the study. Apostolos Gkatzionis and Eleanor Sanderson counselled on methodology. Helene Gellert‐Kristensen, Stefan Stender, and Apostolos Gkatzionis identified relevant summary statistics and performed analyses. Helene Gellert‐Kristensen, Stefan Stender, George Davey Smith, Apostolos Gkatzionis, and Eleanor Sanderson interpreted the results. Helene Gellert‐Kristensen drafted the manuscript and created the figures and tables. Helene Gellert‐Kristensen, Stefan Stender, George Davey Smith, Apostolos Gkatzionis, and Eleanor Sanderson reviewed and commented on the manuscript.

## Funding

This work was supported by Danmarks Frie Forskningsfond, 9060‐00012B. Novo Nordisk Fonden, NNF22OC0075038.

## Consent

We used data from published articles with permission from the authors and publicly available data. The data sources and their origin are described in the manuscripts. All studies underlying the data have ethical approval and informed patient consent. More detailed descriptions are in the original sources.

## Conflicts of Interest

The authors declare no conflicts of interest.

## Supporting information


**Data S1:** Supporting Information.

## Data Availability

The data that support the findings of this study are available from the corresponding author upon reasonable request.
