# Supplementary material for: Disentangling the Effect of BMI on Hepatocellular Carcinoma From Cirrhosis With Multivariable Mendelian Randomization
Source: Liver Int. 2026 Mar 19;46(4):e70609. doi: 10.1111/liv.70609 (PMC13000872; doi:10.1111/liv.70609)
Supplement: Supplementary file 1 — Data S1: Supporting Information. [file LIV-46-0-s001.docx]

**Supplemental Material**

**Disentangling the Effect of BMI on Hepatocellular Carcinoma from Cirrhosis with Multivariable Mendelian Randomization.**

Apostolos Gkatzionis^1^, Eleanor Sanderson^1^, George Davey Smith^1^, Stefan Stender^2,3^, Helene Gellert-Kristensen^2^

^1^MRC Integrative Epidemiology Unit, Population Health Sciences, University of Bristol, Bristol, United Kingdom; ^2^Department of Clinical Biochemistry, Copenhagen University Hospital – Rigshospitalet, Copenhagen, Denmark; ^3^Department of Clinical Medicine, Faculty of Health and Medical Sciences, University of Copenhagen, Copenhagen, Denmark.

Table of content

[Supplemental Figure 1: Scatter plot of BMI SNPs and their effect on BMI and hepatocellular carcinoma. 3](#_Toc211955613)

[Supplemental Figure 2: Plot of the fitted vs. residual values from the MVMR-IVW analysis using BMI and cirrhosis as exposures and hepatocellular carcinoma as outcome. 4](#_Toc211955614)

[Supplemental Table 1: Table of exposure genetic instruments is included in the article. 5](#_Toc211955615)

[Supplemental Table 2: Univariable Mendelian randomization with BMI as exposure and hepatocellular carcinoma as outcome. 9](#_Toc211955616)

[Supplemental Table 3: MVMR-IVW analysis with BMI and cirrhosis as exposures and hepatocellular carcinoma as outcome. 10](#_Toc211955617)

[Supplemental Table 4: Characteristics of outlier variants in the MVMR-IWV analysis with BMI and cirrhosis as exposures and hepatocellular carcinoma as outcome. 11](#_Toc211955618)

[Supplemental Table 5: MVMR-IVW analysis with BMI and cirrhosis as exposures and hepatocellular carcinoma as outcome excluding outlier variants. 12](#_Toc211955619)

[Supplemental Table 6: MVMR-IVW analyses of BMI, cirrhosis, and additional exposures and hepatocellular carcinoma as outcome. 13](#_Toc211955620)

[Supplemental Table 7: Sensitivity analyses of BMI, cirrhosis, and alcohol intake with hepatocellular carcinoma as outcome MVMR. 16](#_Toc211955621)

[Supplemental Table 8: Univariable Mendelian randomization of time-varying body size and liver disease. 17](#_Toc211955622)

[Supplemental Table 9: MVMR-IVW analysis with childhood and adult body size as exposures and cirrhosis as outcome. 18](#_Toc211955623)

[Supplemental Table 10: MVMR-IVW analysis with childhood and adult body size as exposures and hepatocellular carcinoma as outcome. 19](#_Toc211955624)

[Supplemental Table 11: MVMR-IVW analysis with childhood body size, adult body size, and cirrhosis as exposures and hepatocellular carcinoma as outcome. 20](#_Toc211955625)

# Supplemental Figure 1: Scatter plot of BMI SNPs and their effect on BMI and hepatocellular carcinoma.


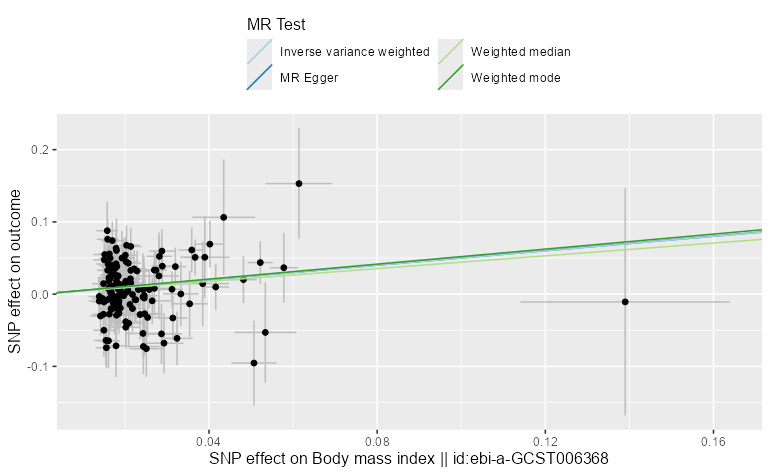


Scatterplot of the 140 SNPs included in the univariable Mendelian randomization of BMI on hepatocellular carcinoma. Each dot represents an SNP plotted as the beta-coefficient (whiskers are SD) for BMI and hepatocellular carcinoma on the X and Y axes, respectively. The lines are the regression lines using the IVW, MR-Egger, weighted median, and weighted mode methods. The SNP rs144839874 had the largest effect on BMI. BMI: body mass index. MR: Mendelian randomization. IVW: inverse variance weighted. SD: standard deviation. SNP: single nucleotide polymorphism.

# Supplemental Figure 2: Plot of the fitted vs. residual values from the MVMR-IVW analysis using BMI and cirrhosis as exposures and hepatocellular carcinoma as outcome.


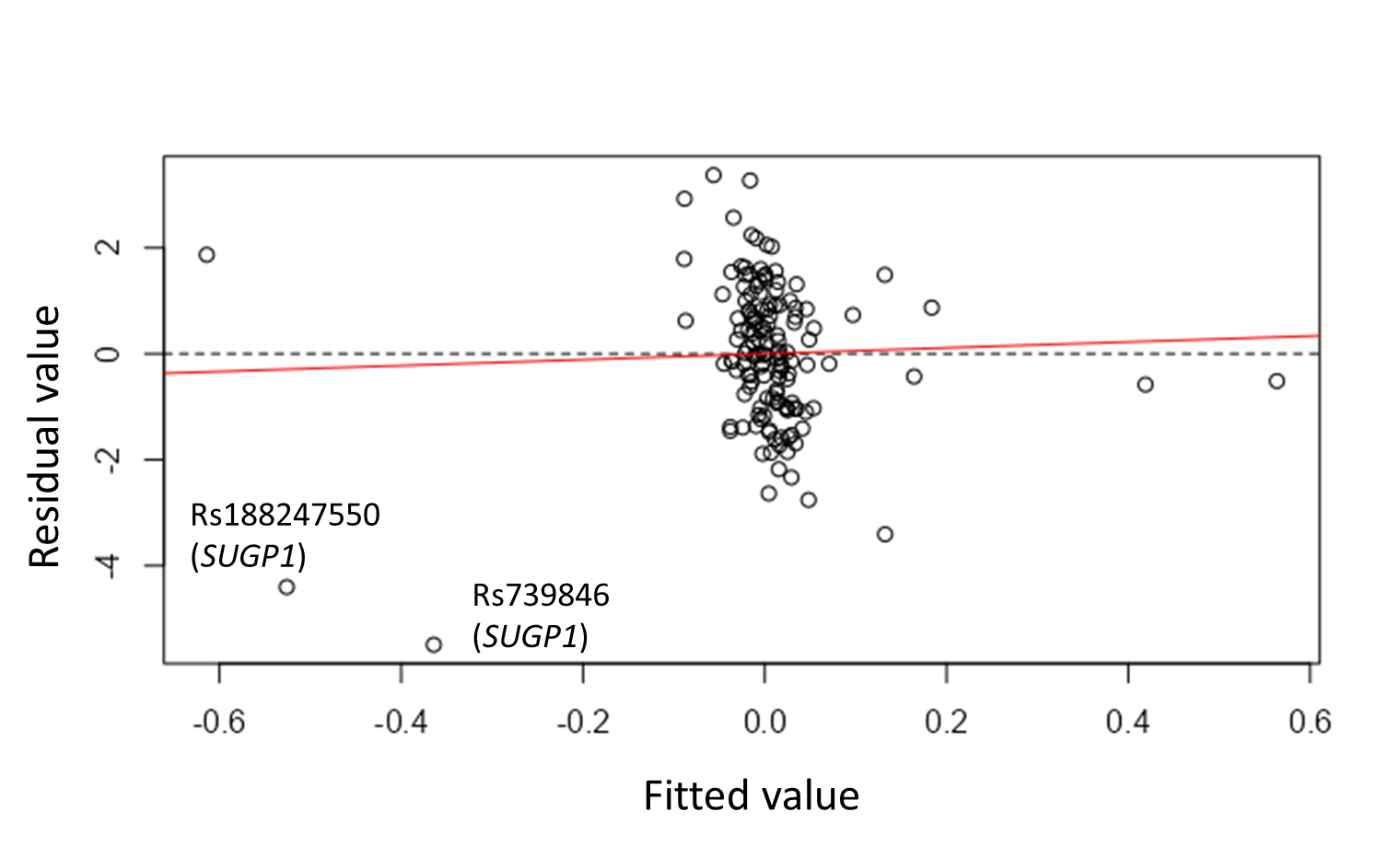


We aggregated the genetic instruments for BMI and cirrhosis into one combined instrument. The genetic instruments were clumped once each and then clumped again when combined based on linkage disequilibrium to attain the genetic instrument for MVMR. We excluded SNPs absent from the exposure or outcome summary statistics. We used the summary statistics and the metaCCA R-package to calculate the phenotypic covariance matrix for the exposures to account for the covariance of the original genetic instruments. BMI: body mass index. MVMR: multivariable Mendelian randomization. IVW: inverse-variance weighted. SNP: single nucleotide polymorphism. SUGP1: SURP and G-patch domains-containing protein 1.

# Supplemental Table 1: Table of exposure genetic instruments is included in the article.

| **Exposure** | **Unit** | **N total or case/controls** | **N SNPs in instrument** | **Pubmed ID** | **OpenGWAS identifier** | **Full summary statistics available** | **Notes** |
| --- | --- | --- | --- | --- | --- | --- | --- |
| Body mass index (BMI) | Z-score | 315,347 | 152 | 30108127 | ebi-a-GCST006368 | Yes | Meta-analysis of Europeans in GERA and Locke 2015 |
| Cirrhosis | Log(odds) | 15,225/1,564,786 | 11 | 38632349 | Not available | Yes | ICD codes |
| Alcohol | Drinks per week | 2,428,851 | 98 | 36477530 | Not available | Yes |  |
| C-reactive protein (CRP) | Ln(mg/l) | 575,531 | 264 | 35459240 | ebi-a-GCST90029070 | No | High sensitivity CRP |
| Interleukin-1-receptor antagonist (IL-1R antagonist) | Standardized rank-based, inverse-normal transformed(log2(NPX)) | 21,758 | 4 | 33067605 | ebi-a-GCST90012004 | No | Measured with Olink |
| Interleukin-6 (IL-6) |  |  | 2 |  | ebi-a-GCST90012005 | No |  |
| Interleukin-6 receptor subunit alpha (IL-6R subunit alpha) |  |  | 7 |  | ebi-a-GCST90012025 | No |  |
| Waist-to-hip ratio | Quantile normalized(ratio) | 502,773 | 317 | 34226706 | ebi-a-GCST90029009 | No | Transformation of variable described in PMID: 29892013 |
| Diabetes mellitus type II (diabetes) | Log(odds ratio) | 62,892/596,424 | 118 | 30054458 | ebi-a-GCST006867 | Yes | Meta-analysis of GERA, DIAGRAM, and UK Biobank |
| Hemoglobin A1C (HbA1C) | Z-score | 389,889 | 404 | 34017140 | ebi-a-GCST90014006 | No | Rank-inverse-based normal transformation of original mmol/mol measurement |
| Homeostasis model assessment of β-cell function (HOMA-B) | Log(HOMA-B) | 36,466 | 4 | 20081858 | ieu-b-117 | Yes | Inverse variance-weighted meta-analyses of GWAS from MAGIC consortium cohorts |
| Childhood body size | Category increase* | 453,169 | 206 | 32376654 | ieu-b-5107 | Yes | Categories of “plumper, thinner or about average”. Validated against measured BMI at mean age 10, 18 in the Avon Longitudinal Study of Parents and Children and BMI at age 12-15 in the Norwegian Trøndelag Health Study. |
| Adult body size | Category increase* |  | 339 |  | ieu-b-5118 | Yes | BMI in adulthood was converted to categories of “plumper, thinner or about average” to match childhood body size. |

OpenGWAS identifier is a unique identifier for each Genome-wide association study available through the “ieu open gwas project” website (<https://gwas.mrcieu.ac.uk/>). The genetic instrument for childhood body size was derived from a genome-wide association study of childhood body size and has been validated against childhood BMI in two separate cohorts. The genetic instrument for adult body size is based on BMI measurements, which were converted to body size categories before the genome-wide association analysis. These genetic instruments have been used to separate childhood body size from adult body size in previous studies. *The body size genetic instruments, ieu-b-5118 and ieu-b-5107, are registered in units of SD and km/m^2^ on the “ieu open gwas project” website, however, the results are indeed in category increase.

# Supplemental Table 2: Univariable Mendelian randomization with BMI as exposure and hepatocellular carcinoma as outcome.

| **Method** | **Beta estimate** | **Standard error** | **P-value** | **Intercept beta** | **Intercept P-value** |
| --- | --- | --- | --- | --- | --- |
| Inverse variance weighted | 0.4999282 | 0.1284946 | 9.997361e-05 |  |  |
| Weighted median | 0.4418024 | 0.1835033 | 1.605778e-02 |  |  |
| Weighted mode | 0.5193882 | 0.3374668 | 1.260583e-01 |  |  |
| MR-Egger | 0.5011954 | 0.3892704 | 2.000664e-01 | -2.988525e-05 | 0.9972522 |

The genetic instrument was clumped based on linkage disequilibrium when more than one variant existed within a 10,000 base pair window. 140 instruments were included in the analyses. BMI: body mass index. MR: Mendelian randomization.

Supplemental Table 3: MVMR-IVW analysis with BMI and cirrhosis as exposures and hepatocellular carcinoma as outcome.

| **Exposure** | **N SNPs** | **Conditional F-statistic** | **Qa-statistic P-value for pleiotropy** | **Beta estimate for hepatocellular carcinoma** | **Standard error** | **P-value** |
| --- | --- | --- | --- | --- | --- | --- |
| BMI | 134 | 58 | 0.001008443 | 0.1162341 | 0.14899741 | 4.366164e-01 |
| Cirrhosis | 11 | 16 |  | 1.2305506 | 0.05970243 | 1.162631e-44 |

We aggregated the genetic instruments for BMI and cirrhosis into one combined instrument. The genetic instruments were clumped once each and then clumped again when combined based on linkage disequilibrium to attain the genetic instrument for MVMR. We excluded SNPs absent from the exposure or outcome summary statistics. We used the summary statistics and the metaCCA R-package to calculate the phenotypic covariance matrix for the exposures to account for the covariance of the original genetic instruments. BMI: body mass index. MVMR: multivariable Mendelian randomization. IVW: inverse-variance weighted.

Supplemental Table 4: Characteristics of outlier variants in the MVMR-IWV analysis with BMI and cirrhosis as exposures and hepatocellular carcinoma as outcome.

| **Rs-number** | **Effect allele** | **Effect allele frequency (1000G)** | **Beta estimate for body mass index (Standard error)** | **Beta estimate for cirrhosis (Standard error)** | **Beta estimate for hepatocellular carcinoma (Standard error)** |
| --- | --- | --- | --- | --- | --- |
| rs739846 | G | 0.93 | 0.01130 (0.00523) | -0.2970 (0.0206) | -0.5844 (0.0401) |
| rs188247550 | C | 0.99 | -0.00761 (0.01420) | -0.4267 (0.0365) | -0.8611 (0.0761) |

Variants identified as outliers in Supplemental Figure 1. Both variants are located in the vicinity of *SUGP1.* The reported affect allele frequencies are from the European ancestry population of the 1000Genome Phase 3 extracted via ensemble (<https://www.ensembl.org/index.html>). BMI: body mass index. MVMR: multivariable Mendelian randomization. IVW: inverse-variance weighted. SUGP1: SURP and G-patch domains-containing protein 1.

Supplemental Table 5: MVMR-IVW analysis with BMI and cirrhosis as exposures and hepatocellular carcinoma as outcome excluding outlier variants.

| **Exposure** | **N SNPs** | **Conditional F-statistic** | **Qa-statistic P-value for pleiotropy** | **Beta estimate for hepatocellular carcinoma** | **Standard error** | **P-value** |
| --- | --- | --- | --- | --- | --- | --- |
| BMI | 134 | 58 | 0.07021631 | 0.1722356 | 0.13254313 | 1.959040e-01 |
| Cirrhosis | 9 | 14 |  | 1.0793615 | 0.05803987 | 9.089012e-40 |

Variants identified as outliers in Supplemental Figure 1 (rs739846 and rs188247550) were removed from the analysis before conducting the MVMR-IVW analysis with BMI and cirrhosis as exposures and hepatocellular carcinoma as outcome. We aggregated the genetic instruments for BMI and cirrhosis into one combined instrument. The genetic instruments were clumped once each and then clumped again when combined based on linkage disequilibrium to attain the genetic instrument for MVMR. We excluded SNPs absent from the exposure or outcome summary statistics. We used the summary statistics and the metaCCA R-package to calculate the phenotypic covariance matrix for the exposures to account for the covariance of the original genetic instruments. BMI: body mass index. MVMR: multivariable Mendelian randomization. IVW: inverse-variance weighted.

# Supplemental Table 6: MVMR-IVW analyses of BMI, cirrhosis, and additional exposures and hepatocellular carcinoma as outcome.

| **Exposure** | | **N SNPs** | | | **Conditional F-statistic** | | | | **Qa-statistic P-value for pleiotropy** | **Beta estimate for hepatocellul-ar carcinoma** | **Standard error** | | **P-value** | | |
| --- | --- | --- | --- | --- | --- | --- | --- | --- | --- | --- | --- | --- | --- | --- | --- |
| **Alcohol** | | | | | | | | | | | | | | | |
| BMI | | 118 | | | 42 | | | | 0.004273444 | 0.0944527 | 0.14819933 | | 5.247028e-01 | | |
| Alcohol | | 66 | | | 20 | | | |  | -0.8111402 | 0.31094750 | | 9.842428e-03 | | |
| Cirrhosis | | 9 | | | 12 | | | |  | 1.1875522 | 0.05687608 | | 2.645046e-50 | | |
| **C-reactive protein (CRP)** | | | | | | | | | | | | | | | |
| BMI | | 87 | | | 23 | | | | 0.02732719 | 0.17527798 | 0.15500312 | | 2.591269e-01 | | |
| CRP | | 201 | | | 102 | | | |  | -0.01430991 | 0.07689989 | | 8.525159e-01 | | |
| Cirrhosis | | 9 | | | 9.0 | | | |  | 1.19869249 | 0.05228686 | | 1.130986e-65 | | |
| **Interleukin-1-receptor antagonist (****IL-1R antagonist)** | | | | | | | | | | | | | | | |
| BMI | | 134 | | | 29 | | | | 0.00108781 | 0.111770476 | 0.15151191 | | | | 4.619050e-01 |
| IL-1R antagonist | | 2 | | | 5 | | | |  | -0.004441014 | 0.12470674 | | | | 9.716417e-01 |
| Cirrhosis | | 10 | | | 13 | | | |  | 1.220771376 | 0.06068009 | | | | 1.536771e-43 |
| **Interleukin-6 (IL-6)** | | | | | | | | | | | | | | | |
| BMI | | 133 | | | 30 | | | | 0.0008314341 | 0.12502764 | 0.15579848 | | 4.235978e-01 | | |
| IL-6 | | 2 | | | 2.5 | | | |  | -0.03785587 | 0.15357807 | | 8.056544e-01 | | |
| Cirrhosis | | 11 | | | 10 | | | |  | 1.22801950 | 0.06003995 | | 2.649614e-44 | | |
| **Interleukin-6 receptor subunit alpha levels (****IL-6R subunit alpha)** | | | | | | | | | | | | | | | |
| BMI | 133 | | | | | 56 | | | 0.001154134 | 0.1067586446 | | 0.14850205 | | 4.733416e-01 | |
| IL-6R subunit alpha | 6 | | | | | 12 | | |  | 0.0001295071 | | 0.08484872 | | 9.987842e-01 | |
| Cirrhosis | 11 | | | | | 15 | | |  | 1.2289129000 | | 0.05949688 | | 2.624711e-45 | |
| **Waist-to-hip ratio** | | | | | | | | | | | | | | | |
| BMI | | | | 81 | | | | 20 | 0.1101597 | 0.14182414 | 0.1443487 | | 3.265883e-01 | | |
| Waist-to-hip ratio | | | | 239 | | | | 36 |  | 0.07043978 | 0.1162745 | | 5.450716e-01 | | |
| Cirrhosis | | | | 8 | | | | 6.7 |  | 1.13982928 | 0.0538322 | | 6.884300e-63 | | |
| **Diabetes mellitus type II (diabetes)** | | | | | | | | | | | | | | | |
| BMI | | | 100 | | | | 35 | | 0.002942854 | 0.13349522 | 0.16420206 | | 4.173264e-01 | | |
| Diabetes type II | | | 79 | | | | 33 | |  | 0.01447483 | 0.05410456 | | 7.893728e-01 | | |
| Cirrhosis | | | 6 | | | | 11 | |  | 1.16077028 | 0.06176528 | | 7.725836e-44 | | |
| **Hemoglobin A1C (****HbA1C)** | | | | | | | | | | | | | | | |
| BMI | | | 58 | | | | 16 | | 0.0001507926 | 0.2196205 | 0.16467890 | | 1.831735e-01 | | |
| HbA1C | | | 304 | | | | 93 | |  | 0.0214884 | 0.08191322 | | 7.932169e-01 | | |
| Cirrhosis | | | 7 | | | | 3.7 | |  | 1.0758396 | 0.07424377 | | 9.086149e-38 | | |
| **Homeostasis model assessment of β-cell function (HOMA-B)** | | | | | | | | | | | | | | | |
| BMI | | | 121 | | | | 18 | | 0.007980022 | 0.09072066 | 0.16050698 | | 5.729259e-01 | | |
| HOMA-B | | | 3 | | | | 3.4 | |  | 0.48283614 | 0.48345859 | | 3.198331e-01 | | |
| Cirrhosis | | | 6 | | | | 14 | |  | 1.20983205 | 0.06370431 | | 6.440654e-39 | | |

We aggregated the genetic instruments for BMI, cirrhosis, and the additional exposure into one combined instrument. The genetic instruments were clumped once each and then clumped again when combined based on linkage disequilibrium to attain the genetic instrument for MVMR. We excluded SNPs absent from the exposure or outcome summary statistics. We used the summary statistics and the metaCCA R-package to calculate the phenotypic covariance matrix for the exposures in the models containing alcohol or diabetes mellitus type II to account for the covariance of the original genetic instruments. For the other models the covariance was set to zero. BMI: body mass index. CRP: C-reactive protein. HbA1c: Hemoglobin A1c. HOMA-B: homeostatic model assessment of beta-cell function. IL-1R: interleukin-1 receptor. IL-6R: Interleukin-6 receptor. IVW: inverse variance-weighted. MVMR: multivariable Mendelian randomization.

# Supplemental Table 7: Sensitivity analyses of BMI, cirrhosis, and alcohol intake with hepatocellular carcinoma as outcome MVMR.

| **Exposure** | **Beta estimate for hepatocellular carcinoma** | **Standard error** | **Odds ratio (95% CI)** |
| --- | --- | --- | --- |
| **MVMR Median** | | | |
| BMI | 0.1143639 | 0.14819933 | 1.12 (0.78 – 1.60) |
| Alcohol | -0.5320856 | 0.4088024 | 0.59 (0.26 – 1.31) |
| Cirrhosis | 1.1114088 | 0.1511265 | 3.04 (2.26 – 4.09) |
| **MVMR Robust** | | | |
| BMI | 0.2518361 | 0.1825828 | 1.29 (0.97 – 1.71) |
| Alcohol | -0.3120352 | 0.3676059 | 0.73 (0.36 – 1.50) |
| Cirrhosis | 0.6665992 | 0.2360907 | 1.95 (1.23 – 3.09) |

We aggregated the genetic instruments for BMI, cirrhosis, and alcohol into one combined instrument. The genetic instruments were clumped once each and then clumped again when combined based on linkage disequilibrium to attain the genetic instrument for MVMR. We excluded SNPs absent from the exposure or outcome summary statistics. BMI: body mass index. CI: confidence interval. IVW: inverse variance-weighted. MVMR: multivariable Mendelian randomization.

# Supplemental Table 8: Univariable Mendelian randomization of time-varying body size and liver disease.

| **Exposure** | **Outcome** | **N SNPs** | **Q-stat** | **F-stat** | **Egger-intercept P-value for Pleiotropy** | **Beta estimate** | **Standard error** | **P-value** |
| --- | --- | --- | --- | --- | --- | --- | --- | --- |
| Childhood body size | Cirrhosis | 211 | 358 | 76 | 0.5264209 | 0.2843683 | 0.09106443 | 0.001791909 |
| Childhood body size | Hepatocellular carcinoma | 195 | 242 | 76 | 0.1822246 | 0.5774007 | 0.1718679 | 0.0007806649 |
| Adult body size | Cirrhosis | 333 | 481 | 59 | 0.5692177 | 0.6261676 | 0.07731985 | 5.568337e-16 |
| Adult body size | Hepatocellular carcinoma | 313 | 357 | 59 | 0.245899 | 0.7559029 | 0.1510362 | 5.592555e-07 |

The analyses were multiplicative random effects meta-analysis weighted by the inverse of the standard error of the SNP-specific exposure-outcome causal effect estimate squared and with correction for under-dispersion – the default inverse variance-weighted (IVW) analysis in the TwoSampleMR package at the time of this study. The genetic instrument for childhood body size is derived from a genome-wide association study of childhood body size and has been validated against childhood BMI in two separate cohorts. The genetic instrument for adult body size is based on BMI measurements, which were converted to body size categories for the genome-wide association study. It has been used to separate childhood body size from adult body size in previous studies. The genetic instrument was clumped based on linkage disequilibrium when more than one variant existed within a 10,000 base pair window. The P-value of the intercept from the MR-Egger method was used to indicate horizontal pleiotropy in the genetic instruments. BMI: body mass index. MR: Mendelian randomization. SNP: single nucleotide polymorphism. Stat: statistic.

# Supplemental Table 9: MVMR-IVW analysis with childhood and adult body size as exposures and cirrhosis as outcome.

| **Exposure** | **N SNPs** | **Conditional F-statistic** | **Qa-statistic P-value for pleiotropy** | **Beta estimate for Cirrhosis** | **Standard error** | **P-value** |
| --- | --- | --- | --- | --- | --- | --- |
| Childhood body size | 138 | 15 | 1.463876e-08 | -0.2611417 | 0.1361948 | 5.599679e-02 |
| Adult body size | 278 | 17 |  | 0.7998095 | 0.1214937 | 1.680908e-10 |

We aggregated the genetic instruments for childhood and adult BMI into one combined instrument. The genetic instrument for childhood body size is derived from a genome-wide association study of childhood body size and has been validated against childhood BMI in two separate cohorts. The genetic instrument for adult body size is based on BMI measurements, which were converted to body size categories for the genome-wide association study. It has been used to separate childhood BMI from adult BMI in previous studies. The genetic instruments were clumped once each and then clumped again when combined based on linkage disequilibrium to attain the genetic instrument for MVMR. We excluded SNPs absent from the exposure or outcome summary statistics. We used the summary statistics and the metaCCA R-package to calculate the phenotypic covariance matrix for the exposures to account for the covariance of the original genetic instruments. BMI: body mass index. MVMR: multivariable Mendelian randomization. IVW: inverse-variance weighted. SNP: single nucleotide polymorphism.

# Supplemental Table 10: MVMR-IVW analysis with childhood and adult body size as exposures and hepatocellular carcinoma as outcome.

| **Exposure** | **N SNPs** | **Conditional F-statistic** | **Qa-statistic P-value for pleiotropy** | **Beta estimate for hepatocellular carcinoma** | **Standard error** | **P-value** |
| --- | --- | --- | --- | --- | --- | --- |
| Childhood body size | 134 | 15 | 0.04769194 | 0.09661804 | 0.2582979 | 0.7085960727 |
| Adult body size | 268 | 17 |  | 0.78304532 | 0.2314093 | 0.0007985941 |

We aggregated the genetic instruments for childhood and adult BMI into one combined instrument. The genetic instrument for childhood body size is derived from a genome-wide association study of childhood body size and has been validated against childhood BMI in two separate cohorts. The genetic instrument for adult body size is based on BMI measurements, which were converted to body size categories for the genome-wide association study. It has been used to separate childhood body size from adult body size in previous studies. The genetic instruments were clumped once each and then clumped again when combined based on linkage disequilibrium to attain the genetic instrument for MVMR. We excluded SNPs absent from the exposure or outcome summary statistics. We used the summary statistics and the metaCCA R-package to calculate the phenotypic covariance matrix for the exposures to account for the covariance of the original genetic instruments. BMI: body mass index MVMR: multivariable Mendelian randomization. IVW: inverse-variance weighted. SNP: single nucleotide polymorphism.

# Supplemental Table 11: MVMR-IVW analysis with childhood body size, adult body size, and cirrhosis as exposures and hepatocellular carcinoma as outcome.

| **Exposure** | **N SNPs** | **Conditional F-statistic** | **Qa-statistic P-value for pleiotropy** | **Beta estimate for hepatocellular carcinoma** | **Standard error** | **P-value** |
| --- | --- | --- | --- | --- | --- | --- |
| Childhood body size | 136 | 12 | 0.08451008 | 0.4188182 | 0.29136231 | 0.1515186 |
| Adult body size | 253 | 13 |  | -0.1903734 | 0.26595918 | 0.4746110 |
| Cirrhosis | 10 | 7.6 |  | 1.1617410 | 0.04973431 | 1.916587e-72 |

We aggregated the genetic instruments for childhood and adult BMI into one combined instrument. The genetic instrument for childhood body size is derived from a genome-wide association study of childhood body size and has been validated against childhood BMI in two separate cohorts. The genetic instrument for adult body size is based on BMI measurements, which were converted to body size categories for the genome-wide association study. It has been used to separate childhood body size from adult body size in previous studies. The genetic instruments were clumped once each and then clumped again when combined based on linkage disequilibrium to attain the genetic instrument for MVMR. We excluded SNPs absent from the exposure or outcome summary statistics. We used the summary statistics and the metaCCA R-package to calculate the phenotypic covariance matrix for the exposures to account for the covariance of the original genetic instruments. BMI: body mass index MVMR: multivariable Mendelian randomization. IVW: inverse-variance weighted. SNP: single nucleotide polymorphism.
